# COL3A1 and SNAP91: novel glioblastoma markers with diagnostic and prognostic value

**DOI:** 10.18632/oncotarget.12038

**Published:** 2016-09-15

**Authors:** Yuan-Feng Gao, Xiao-Yuan Mao, Tao Zhu, Chen-Xue Mao, Zhi-Xiong Liu, Zhi-Bin Wang, Ling Li, Xi Li, Ji-Ye Yin, Wei Zhang, Hong-Hao Zhou, Zhao-Qian Liu

**Affiliations:** ^1^ Department of Clinical Pharmacology, Xiangya Hospital, Central South University, Changsha 410008, P. R. China; ^2^ Institute of Clinical Pharmacology, Central South University, Hunan Key Laboratory of Pharmacogenetics, Changsha 410078, P. R. China; ^3^ Department of Neurosurgery, Xiangya Hospital, Central South University, Changsha 410008, P. R. China

**Keywords:** glioblastoma, novel glioblastoma markers, prognostic value, COL3A1, SNAP91

## Abstract

Although patients with glioblastoma (GBM) have grave prognosis, significant variability in patient outcome is observed. This study aims to identify novel targets for GBM diagnosis and therapy. Microarray data (GSE4290, GSE7696, and GSE4412) obtained from the Gene Expression Omnibus was used to identify the differentially expressed genes (DEGs) by significant analysis of microarray (SAM). Intersection of the identified DEGs for each profile revealed 46 DEGs in GBM. A subset of common DEGs were validated by real-time reverse transcription quantitative PCR (qPCR). The prognostic value of some of the markers was also studied. We determined that *RRM2* and *COL3A1* were increased and directly correlated with glioma grade, while *SH3GL2 and SNAP91* were decreased in GBM and inversely correlated with glioma grade. Kaplan-Meir analysis of GSE7696 revealed that *COL3A1* and *SNAP91* correlated with survival, suggesting that *COL3A1* and *SNAP91* may be suitable biomarkers for diagnostic or therapeutic strategies for GBM.

## INTRODUCTION

Glioma is the most common malignant primary brain tumor in humans, occurring in 6 of every 100,000 people. With a five-year survival rate of 20-30%, it is also one of the most aggressive [1]. These tumors are composed of a heterogeneous population of cells that contributes to the resiliency of the disease [2]. Generally speaking, gliomas are classified as either relatively slow growing low-grade (I or II) tumors or rapidly growing, highly metastatic high-grade (III or IV) tumors [3]. Overall, the disease is a fast-progressing fatal malignancy and the majority of patients with high-grade gliomas (III or IV) suffer from a poor quality of life [4, 5]. Currently, the standard clinical treatment is surgical resection followed by radiotherapy and chemotherapy [6–9]. However, patients who receive these treatments may develop resistance to chemotherapy [10]. Thus, recent efforts have focused on identification of candidate biomarkers of glioblastoma (GBM; grade IV glioma) development for early detection and to produce more effective therapeutic strategies [11–13].

At present, diagnosis of GBMs is mainly based on histological detection. Although the importance of numerous genes, including EGFR, bFGF, VEGF, IGF-1, p53 and p16 [14–19] to glioma progression has been well established. These genes are neither predictive of survival of glioma patients nor able to guide therapeutic decisions. With the continuous development of biotechnology and the innovation of novel high-throughput technology, studies have begun to investigate diseases at the genome level, and gene chip technology has become more common. To date, microarray analysis has been successfully used to identify unknown glioma-associated oncogenes [20], and analyze gene expression within different biological networks [21–23].

In the present study, we analyzed three microarray gene expression profiles to examine changes in gene expression associated with glioma progression and identify novel targets for glioblastoma diagnosis and therapy. We identified 46 differentially expressed genes in GBM that were common among all three profiles. Differential expression of a subset of differentially expressed genes (DEGs) was validated by real-time quantitative reverse transcription PCR (qPCR). We determined that *RRM2* and *COL3A1* were up-regulated and directly correlated with glioma grade, while *SH3GL2 and SNAP91* were down-regulated in GBM and inversely correlated with glioma grade. Kaplan-Meir analysis of GSE7696 revealed that *COL3A1* and *SNAP91* correlated with survival, suggesting that *COL3A1* and *SNAP91* may be suitable biomarkers for diagnostic or therapeutic strategies for GBM.

## RESULTS

### Identification of DEGs

**Figure 1:**
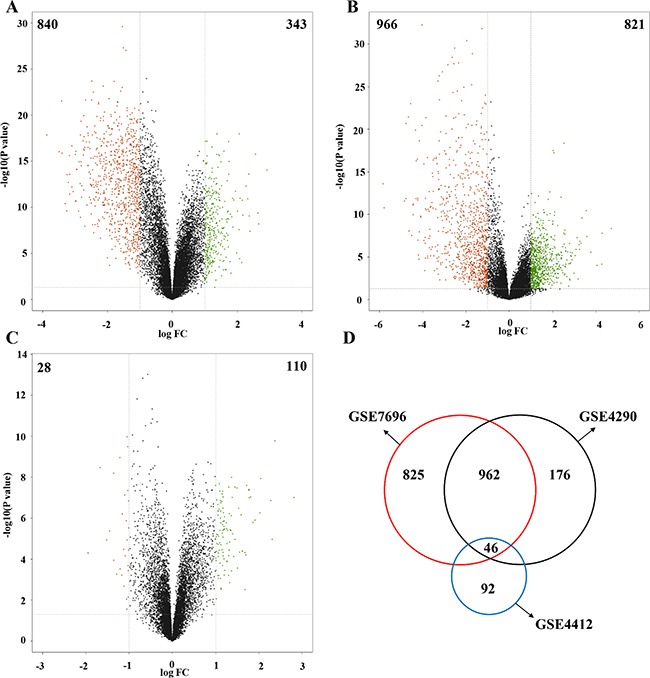
Identification of DEGs **A.** There were 1,183 genes between normal and tumor tissues in GSE4290 were filtered as DEGs, including 343 up-regulated and 840 down-regulated genes. **B.** A total of 1,787 genes between normal and tumor tissues in GSE7696 were filtered as DEGs, including 821 up-regulated and 966 down-regulated genes. **C.** 138 genes between grade IV and III grade samples in GSE4412 were filtered as DEGs, including 110 up-regulated and 28 down-regulated genes. **D.** After intersection, a total of 46 DEGs were detected.

Three gene expression profiles (GSE4290, GSE7696 and GSE4412) of non-tumor, low grade glioma and high grade glioma tissue samples were analyzed to identify genes differentially expressed during tissue progression. A total of 1,183 genes (343 up-regulated and 840 down-regulated genes) between normal and tumor tissues in GSE4290, 1,787 genes (821 up-regulated and 966 down-regulated genes) between normal and GBM tissues in GSE7696, and 138 genes (110 up-regulated and 28 down-regulated genes) between grade IV and III grade glioma samples in GSE4412 were filtered as differentially expressed genes (DEGs) (Figure 1A–1C, Supplementary Table S1). Intersection of the DEGs identified a total of 46 common DEGs, suggesting that these DEGs may play an important role during glioma progression (Figure 1D, Table 1). Moreover, the results of cluster analysis are showed in Supplementary Figure S1.

**Table 1:** Differently expressed genes (DEGs) in glioblastoma tissue samples

	Differently Expressed Genes (DEGs)
Upregulated	RRM2, COL3A1, COL1A1, COL4A1, TNC, COL4A2, PTX3, ANXA1, VEGFA, NNMT, CHI3L1, COL1A2, GBP1, LAMB1, IGFBP2, IGFBP3, TUBB6, PROS1, TGFBI, C1R, FSTL1, EMP3, ANXA2P2, SLC2A10, S100A10, FCGBP, SERPINA3, CLIC1, TRIP6, SERPINH1, C1QB, S100A11, CPVL, CFI, HLA-DRB1, C1QA
Downregulated	SH3GL2, SNAP91, MOBP, ETNPPL, GABBR2, ATP6V1G2, HLF, SLITRK5, GNAO1, NTSR2

Note : RRM2, ribonucleotide reductase M2; COL3A1, collagen, type III, alpha 1; COL1A1, collagen, type I, alpha 1; COL4A1, collagen, type IV, alpha 1; TNC, tenascin C; COL4A2, collagen, type IV, alpha 2; PTX3, pentraxin 3; ANXA1, annexin A1; VEGFA, vascular endothelial growth factor A; NNMT, nicotinamide N-methyltransferase; CHI3L1, chitinase 3-like 1; COL1A2, collagen, type I, alpha 2; GBP1, guanylate binding protein 1; LAMB1, laminin, beta 1; IGFBP2, insulin-like growth factor binding protein 2; IGFBP3, insulin-like growth factor binding protein 3; TUBB6, tubulin, beta 6; PROS1, protein S1; TGFBI, transforming growth factor, beta-induced; C1R, complement component 1; FSTL1, follistatin-like 1; EMP3, epithelial membrane protein 3; ANXA2P2, annexin A2 pseudogene 2; SLC2A10, solute carrier family 2, member 10; S100A10, S100 calcium binding protein A10; FCGBP, Fc fragment of IgG binding protein; SERPINA3, serpin peptidase inhibitor, clade A, member 3; CLIC1, chloride intracellular channel 1; TRIP6, thyroid hormone receptor interactor 6; SERPINH1, serpin peptidase inhibitor, clade H, member 1; C1QB, complement component 1, q subcomponent, B chain; S100A11, S100 calcium binding protein A11; CPVL, carboxypeptidase, vitellogenic-like; CFI, complement factor I; HLA-DRB1, major histocompatibility complex, class II, DR beta 1; C1QA, complement component 1, q subcomponent, A chain; SH3GL2, SH3-domain GRB2-like 2; SNAP91, synaptosomal-associated protein; MOBP, myelin-associated oligodendrocyte basic protein; ETNPPL, ethanolamine-phosphate phospho-lyase; GABBR2, gamma-aminobutyric acid (GABA) B receptor 2; ATP6V1G2, ATPase, V1 subunit G2; HLF, hepatic leukemia factor; SLITRK5, SLIT and NTRK-like family 5; GNAO1, guanine nucleotide binding protein; NTSR2, neurotensin receptor 2.

### Function enrichment of DEGs

To identify the functional differences of DEGs in different glioma grades, gene ontology (GO) and Kyoto Encyclopedia of Genes and Genomes (KEGG) enrichment analyses were performed. Enrichment analysis suggested that DEGs function differed between different glioma grades. DEGs in GBM tissue compared to non-tumor tissue were mainly involved in the regulation of the extracellular matrix (ECM) (Table 2), an environment that is essential to tumor development and maintenance [24, 25].

**Table 2:** Enriched functions of DEGs in glioblastoma (Top 10)

Category	Term	FDR
GOTERM_CC_DIRECT	GO:0005576~extracellular region	2.22E-07
GOTERM_CC_DIRECT	GO:0005615~extracellular space	1.78E-06
GOTERM_MF_DIRECT	GO:0005201~extracellular matrix structural constituent	7.86E-06
GOTERM_CC_DIRECT	GO:0070062~extracellular exosome	1.86E-05
KEGG_PATHWAY	hsa05150:Staphylococcus aureus infection	2.63E-05
GOTERM_CC_DIRECT	GO:0031012~extracellular matrix	3.34E-04
KEGG_PATHWAY	hsa04512:ECM-receptor interaction	6.73E-04
GOTERM_BP_DIRECT	GO:0030198~extracellular matrix organization	0.002918
GOTERM_MF_DIRECT	GO:0048407~platelet-derived growth factor binding	0.003708
KEGG_PATHWAY	hsa04510:Focal adhesion	0.008216

### Independent validation of GBM DEGs

**Figure 2:**
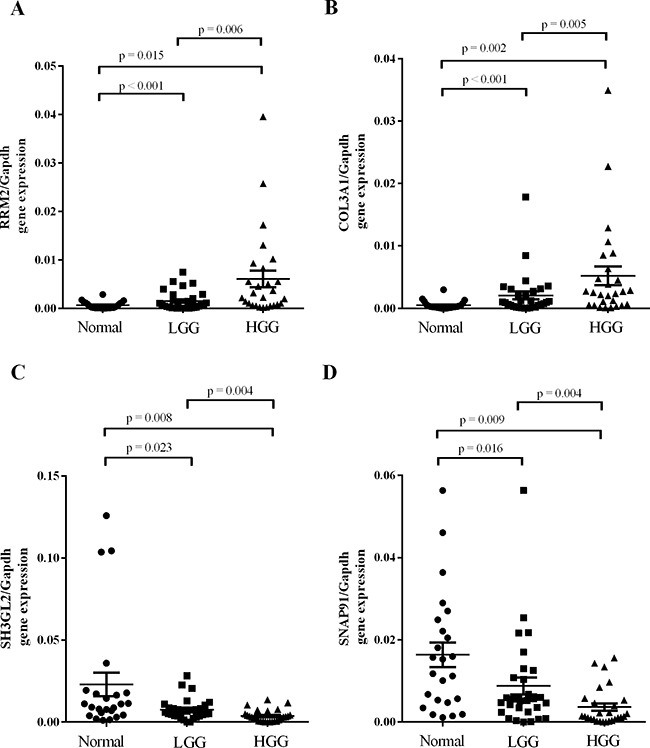
Independent validation of GBM DEGs Some of the relevant genes were validated by real-time quantitative reverse transcription-PCR. **A.** RRM2 and **B.** COL3A1 expression was drastically increased in malignant gliomas and directly correlated with the glioma grade. **C.** SH3GL2 and **D.** SNAP91 expression was drastically decreased in malignant gliomas and also directly correlated with glioma grade.

**Figure 3:**
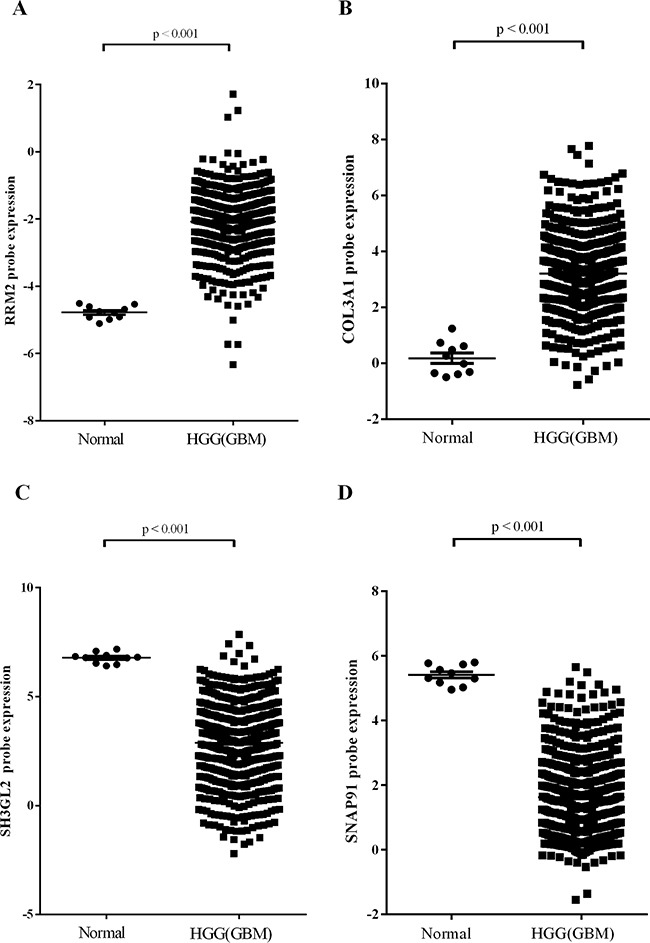
Gene expression in TCGA dataset **A.** RRM2. **B.** COL3A1. **C.** SH3GL2. **D.** SNAP91.

A subset of DEGs in GBM samples compared to non-tumor samples (Table 1) was further validated by real-time quantitative reverse transcription-PCR (qPCR). It's worth noting that there were four genes specifically expressed among DEGs. We found that expression of up-regulated DEGs *RRM2* and *COL3A1* (>2-fold up-regulated as determined by the gene expression profile) was significantly higher in malignant gliomas compared to lower grade gliomas and non-tumor brain tissue and directly correlated with glioma grade (Figure 2A & 2B). Expression of down-regulated DEGs *SH3GL2* and *SNAP91*, which were found to be >2-fold lower in GBM samples by microarray analysis, was lower in malignant gliomas compared to non-tumor tissue and lower grade gliomas. Expression of *SH3GL2* and *SNAP91* also inversely correlated with glioma grade (Figure 2C & 2D). Additionally, the association between gene expression and clinicopathological parameters was analyzed in the present study. No significant association was observed between the expression of validated DEGs and patient age or gender (Table 3, Supplementary Table S2). Gene expression of the validate DEGs was consistent with the TCGA datasets (Figure 3). These results suggest that expression of DEGs correlates with glioma tumor grade.

**Table 3:** Correlation between COL3A1/SNAP91 expression and glioma clinicopathologic features in 57 patients

	N%	COL3A1 expression levels			*P*	SNAP91 expression levels			*P*
		High expression	Low expression	Ratio (High/Low)		High expression	Low expression	Ratio (High/Low)	
Sex									
male	42(73.68)	11	31	0.355	0.278	13	29	0.448	0.930
female	15(26.32)	3	12	0.250		2	13	0.154	
Age, y									
<45	37(64.91)	8	29	0.276	0.419	9	28	0.321	0.903
≥45	20(35.09)	6	14	0.428		6	14	0.428	
Grade									
Low (I + II)	30(52.63)	4	26	0.154	0.050	10	20	0.5	0.031
High (III + IV)	27(47.37)	10	17	0.588		5	22	0.227	

### Survival value of GBM-specific markers

**Figure 4:**
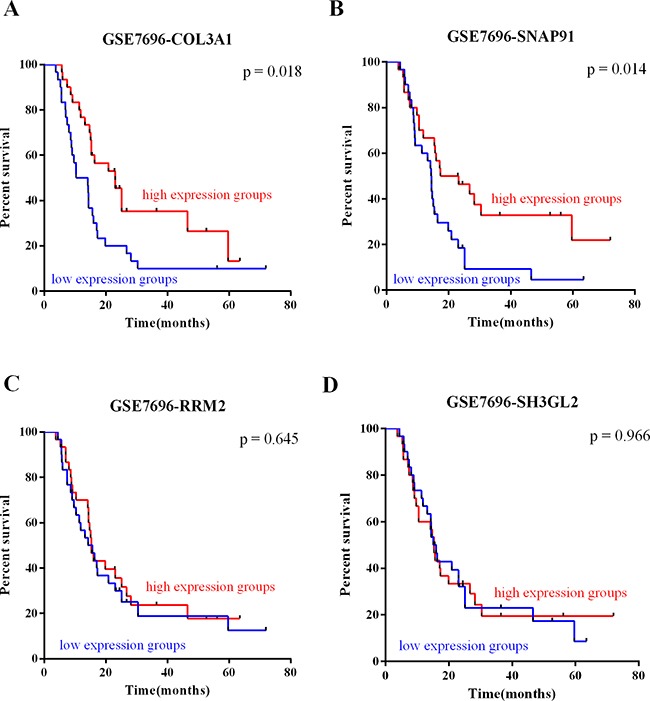
Survival value of GBM-specific markers The survival value of GBM-specific markers was analyzed by subject GSE7696 dataset. In Kaplan-Meier analysis, **A.** COL3A1 and **B.** SNAP91 expression was correlated with survival. However, **C.** RRM2 and **D.** SH3GL2 expression did not correlate with survival.

To investigate the relationship between the expression of the validated DEGs and patient survival, we analyzed the prognostic significance of the genes using Kaplan-Meier analysis for expression profile GSE7696. *COL3A1* and *SNAP91* expression correlated with survival (p = 0.018, Figure 4A; p = 0.014, Figure 4B). These results suggest that high expression of *COL3A1* and *SNAP91* in GBMs confer a survival advantage to patients. However, *RRM2,* and *SH3GL2* expression did not correlate with survival (p = 0.645, Figure 4C; p = 0.966, Figure 4D). Taken together, the data suggest that expression of *COL3A1* and *SNAP91* has prognostic value.

## DISCUSSION

Recently, microarray-based expression profiling studies have revealed that genes differentially expressed among glioma grades could be of prognostic value [26]. Therefore, identification of genes differentially expressed in GBMs compared to lower grade gliomas could greatly facilitate prognostication and our ability to develop effective treatment protocols [27–29]. In order to identify potential biomarkers for glioblastoma prognosis and therapy, we used microarray data (GSE4290, GSE7696, and GSE4412) to identify the differentially expressed genes (DEGs) by significant analysis of microarray (SAM). 46 DEGS were found to be in common in all profiles. A subset of the DEGs identified in GBM—*COL3A1*, *SNAP91, RRM2,* and *SH3GL2*—were also validated by real-time reverse transcription quantitative PCR (qPCR).

A potential prognostic factor for GBM is *COL3A1.* We found that *COL3A1* was up-regulated in GBM tissues. *COL3A1* encodes a fibrillary collagen molecule that has been linked to myocardial infarction [30] and the risk of stroke recurrence and prognosis in Chinese patients [31]. *PDGFRβ* was found to significantly correlate with the reference *COL1A1*, *COL1A2*, and *COL3A1* expression [32]. Another potential target for GBM therapies and diagnosis is *SNAP91.* We determined that *SNAP91* was down-regulated in GMB tissues. *SNAP91* encodes a synapse-associated protein that is expressed highest in the brain [33].

We also determined that *RRM2* and *SH3GL2* are differentially expressed in GBM and could potentially serve as GBM biomarkers. *RRM2* codes an enzyme that's over-expression has been correlated with resistance to radiotherapy and chemotherapy, and enhanced malignant potential in multiple cancers [34–36]. *SH3GL2* is a multifunctional gene that encodes a protein called endophilin-1. Endophilin-1 is primarily distributed in the central nervous system and functions as a tumor suppressor in many tumors. *SH3GL2* has been shown to be decreased by miR-330 and associated with *ERK* and *PI3K/AKT* signaling pathways [37, 38]. We also found that while *RRM2* and *SH3GL2* were differentially expressed in GBM, their expression had no prognostic value for GBM, suggesting that they are tumor-specific, but not GBM specific. Our results are in line with previous studies that showed that *RRM2* was tumor-specific, but not associated with the survival in GBM [39].

Microarray datasets not only renders the analysis of large quantities of biological information quick and simple, but also facilitates the identification of potential biomarkers for various diseases and medical conditions. In this study, we have identified and validated a set of novel GBM biomarker candidate genes. We also provided evidence of the prognostic value of two of these potential markers, *COL3A1* and *SNAP91*. However, further studies are needed to more precisely characterize the functional significance of these genes in glioma progression and their potential prognosis application for glioma needs to receive more attention.

## MATERIALS AND METHODS

### Affymetrix microarray analysis

Three expression profiles (GSE4290, GSE7696, and GSE4412) were acquired from the Gene Expression Omnibus (GEO, http://www.ncbi.nlm.nih.gov/geo/) database. The platform of GSE4290 and GSE7696 is GPL570 [HG-U133_Plus_2] Affymetrix Human Genome U133 Plus 2.0 Array. The platform of GSE4412 is GPL96 [HG-U133A] Affymetrix Human Genome U133A Array. In GSE4290 data, 23 brain tissue samples from epilepsy patients as non-tumor (control) samples and 157 glioma samples, including astrocytoma, oligodendroglioma and glioblastoma samples (45 grade II, 31 grade III and 81grade IV) were used. Total of 80 glioblastoma specimen and 4 non-tumor brain samples were analysis in GSE7696. Only grade III (n = 24) and IV (n = 50) gliomas were included in GSE4412. The original CEL files and probe annotation of the platform was used.

### Data pre-processing

The probe-level data in CEL files were converted into expression profiles. Background correction and quartile data normalization were extracted by the robust multi-array average (RMA) with affy package. For genes corresponding to multiple probe sets, the gene expression values of those sets were averaged [40, 41].

### Differentially expressed genes (DEGs) analysis

Significant analysis of microarray (SAM) using two classes (tumor vs normal) of unpaired measurements was performed for DEGs identification in GSE4290 and GSE7696. GSE4412 only analyzed the DEGs between grade III and grade IV samples. Genes with a |log fold change (FC)| ≥ 2 and p < 0.05 were considered differentially expressed. The results from each analysis were intersected to identify common DEGs that may play a significant role in tumor progression.

### Gene ontology (GO) enrichment analysis

For preliminary investigation into the functional differences of DEGs in glioblastoma (GBM), Database for Annotation Visualization and Integrated Discovery (DAVID) online software (DAVID Bioinformatics Resources 6.7) was used to perform gene ontology (GO) and Kyoto Encyclopedia of Genes and Genomes(KEGG) enrichment analysis. DAVID utilizes Fisher's exact test to enrich functions of certain genes [42].

### Patients and tissue samples

All patients undergoing surgical treatment in the Hunan cancer hospital (Changsha, Hunan, China) for primary brain cancers between 2007 and 2013 were invited to participate in this institutional review board-approved study. This study compiled data for glioma tumor stages I through IV and normal brain controls. Tumors were histopathologically classified according to the WHO classification. The tissue samples were flash frozen in liquid nitrogen immediately after resection and stored at −80 °C for future processing.

### RNA extraction and real-time quantitative PCR

Total RNA was extracted by trizol reagent according to the manufacturer's protocol. When the A260/A280 ratio was between 1.9 and 2.1, the extracted RNA was determined to be pure and was used in subsequent experiments. 2μg RNA was reverse-transcribed using the Primescript RT reagent Kit with gDNA Eraser (Takara Bio Inc, Japan). Real-time PCR was performed using the SYBR Premix DimerEraser kit (Takara Bio Inc, Japan). The reactions were cycled 40 times [95°C, 30 seconds, (95°C, 5 seconds; 55°C, 30 seconds; and 72°C, 30 seconds)] with fluorescence measurements. A melting curve was performed at the end of amplification cycles to verify the specificity of the PCR products. All the determinations were performed in duplicate. Primers used for real-time PCR are shown in Table 4. The relative expression of target gene mRNA was normalized to the expression level of GAPDH mRNA using the 2^−ΔCt^ method.

**Table 4:** Primer sequences used for real-time PCR

Gene	Sequence	Base
RRM2-F	GTGGAGCGATTTAGCCAAGAA	21
RRM2-R	CACAAGGCATCGTTTCAATGG	21
COL3A1-F	TTGAAGGAGGATGTTCCCATCT	22
COL3A1-R	ACAGACACATATTTGGCATGGTT	23
SH3GL2-F	ACTGTCGGAGGTCAAAGACTC	21
SH3GL2-R	CCGGAATCTTGCCTTGTCGTT	21
SNAP91-F	ACCTCCAGCCAGACTTTTCC	20
SNAP91-R	CTCTCCCAAAAGGTCTCCCC	20
GAPDH-F	GAGTCAACGGATTTGGTCGT	20
GAPDH-R	TTGATTTTGGAGGGATCTCG	20

### Statistical analysis

SPSS16.0 software was used for general statistical analyses. Comparisons between two experimental groups were performed using Student's t test. Survival rate was calculated using the Kaplan-Meier method with the log-rank test applied for comparison. All tests performed were two-sided and the criterion for statistical significance was taken as p < 0.05.

## SUPPLEMENTARY FIGURE AND TABLES




