# Supplementary material for: COL3A1 and SNAP91: novel glioblastoma markers with diagnostic and prognostic value
Source: Oncotarget. 2016 Sep 15;7(43):70494–503. doi: 10.18632/oncotarget.12038 (PMC5342568; doi:10.18632/oncotarget.12038)
Supplement: Supplementary file 1 [file oncotarget-07-70494-s001.pdf]

COL3A1 and SNAP91: novel glioblastoma markers with diagnostic and prognostic value

SUPPLEMENTARY FIGURE AND TABLES

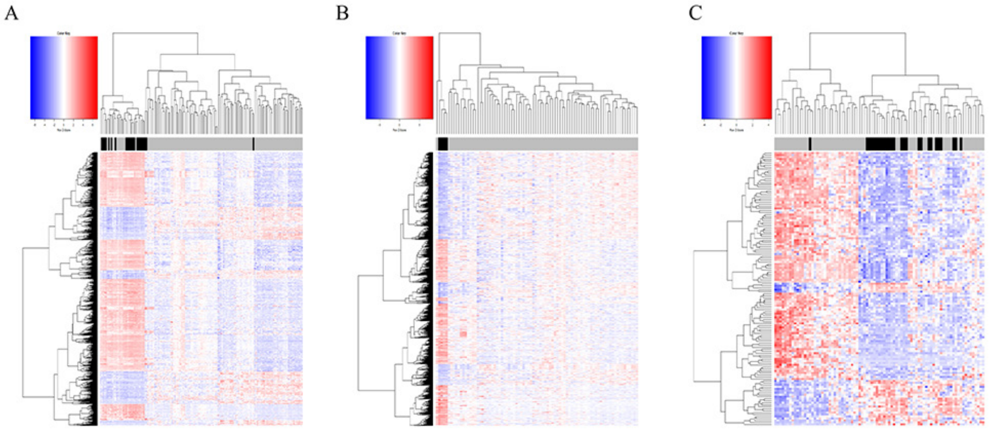

Supplementary Figure S1: Cluster analysis of three datasets. Red and blue reflect high and low expression levels, respectively. **A.** GSE4290. **B.** GSE7696. **C.** GSE4412.

Supplementary Table S1: DEGs.

See Supplementary File 1

Supplementary Table S2: Correlation between RRM2/SH3GL2 expression and glioma clinicopathologic features in 57 patients

	N%	RRM2 expression levels			*P*	SH3GL2 expression levels			*P*
		High expression	Low expression	Ratio (High/Low)		High expression	Low expression	Ratio (High/Low)	
Sex									
male	42(73.68)	11	31	0.355	0.769	17	25	0.680	0.863
female	15(26.32)	6	12	0.250		5	10	0.5	
Age,y									
<45	37(64.91)	12	25	0.480	0.989	14	23	0.609	0.454
≥45	20(35.09)	5	15	0.333		8	12	0.667	
Grade									
Low (I + II)	30(52.63)	5	25	0.2	0.008	16	14	1.142	0.006
High (III + IV)	27(47.37)	12	15	0.8		6	21	0.286	
